# Repeated Methamphetamine Administration Results in Axon Loss Prior to Somatic Loss of Substantia Nigra Pars Compacta and Locus Coeruleus Neurons in Male but Not Female Mice

**DOI:** 10.3390/ijms241713039

**Published:** 2023-08-22

**Authors:** Alexander Pilski, Steven M. Graves

**Affiliations:** Department of Pharmacology, University of Minnesota, Minneapolis, MN 55455, USA; pilsk001@umn.edu

**Keywords:** methamphetamine, neurodegeneration, substantia nigra pars compacta, locus coeruleus, monoamine oxidase, sex difference

## Abstract

Methamphetamine (meth) is a neurotoxic psychostimulant that increases monoamine oxidase (MAO)-dependent mitochondrial oxidant stress in axonal but not somatic compartments of substantia nigra pars compacta (SNc) and locus coeruleus (LC) neurons. Chronic meth administration results in the degeneration of SNc and LC neurons in male mice, and MAO inhibition is neuroprotective, suggesting that the deleterious effects of chronic meth begin in axons before advancing to the soma of SNc and LC neurons. To test this hypothesis, mice were administered meth (5 mg/kg) for 14, 21, or 28 days, and SNc and LC axonal lengths and numbers of neurons were quantified. In male mice, the SNc and LC axon lengths decreased with 14, 21, and 28 days of meth, whereas somatic loss was only observed after 28 days of meth; MAO inhibition (phenelzine; 20 mg/kg) prevented axonal and somatic loss of SNc and LC neurons. In contrast, chronic (28-day) meth had no effect on the axon length or numbers of SNc or LC neurons in female mice. The results demonstrate that repeated exposure to meth produces SNc and LC axonal deficits prior to somatic loss in male subjects, consistent with a dying-back pattern of degeneration, whereas female mice are resistant to chronic meth-induced degeneration.

## 1. Introduction

Methamphetamine (meth) is an addictive psychostimulant with escalating rates of abuse in the United States [1,2,3]. In addition to being highly addictive, meth is also neurotoxic [4,5]. Clinical evidence from human meth users shows decreased dopamine content, tyrosine hydroxylase (TH) and dopamine transporter (DAT) expressions, and vesicular monoamine transporter 2 (VMAT2) and DAT binding in the striatum, suggesting nigrostriatal axon loss [6,7,8,9,10,11,12,13]. Similar outcomes are reported in rodents using acute binge paradigms wherein subjects are administered a single high-dose bolus or multiple injections in a single day, which result in decreased dopamine tissue content; DAT, VMAT2, and TH immunoreactivity; DAT and VMAT2 binding; DAT protein expression; and TH and VMAT2 activity [14,15,16,17,18,19,20,21,22,23,24,25,26,27,28,29,30,31,32,33,34,35]. We have recently reported that the deleterious effects of meth extend beyond nigrostriatal axons. More specifically, chronic (28-day) meth (5 mg/kg; i.p.) administration to male mice results in overt SNc degeneration that is linked to meth-induced axonal mitochondrial oxidant stress [36,37,38].

Meth binds to and induces dysfunction of monoamine reuptake proteins as well as VMAT2. In dopaminergic neurons, the consequence of binding to and inducing dysfunction of VMAT2 is increased cytosolic dopamine concentrations [39,40], which increases mitochondrial oxidant stress in axonal but not somatic subcellular compartments [36,38]. This meth-induced axonal mitochondrial oxidant stress results from MAO metabolism of dopamine, which generates free electrons that are transferred to the mitochondrial intermembrane space [38]. The in vivo administration of meth (5 mg/kg) for 28 consecutive days to male mice results in SNc degeneration (both axonal and somatic), which is prevented by a mitochondrial antioxidant (mitoTEMPO) or a MAO inhibitor [36]. Together, these data suggest that meth-induced MAO-dependent axonal mitochondrial oxidant stress is necessary for degeneration.

In addition to SNc dopamine neurons, meth similarly impacts locus coeruleus (LC) norepinephrine neurons [41]. Like in SNc neurons, meth increases MAO-dependent axonal but not somatic mitochondrial oxidant stress in LC neurons [42]. Furthermore, chronic in vivo administration of meth results in a loss of LC axon length and the number of norepinephrine neurons in the LC of male mice, both of which are prevented by MAO inhibition [42]. Therefore, meth-induced MAO-dependent axonal mitochondrial oxidant stress appears to be necessary for chronic meth-induced LC degeneration, just as in SNc neurons [36,37,42]. However, the manner in which SNc and LC degeneration progresses during chronic meth administration is unclear.

Given that meth increases axonal but not somatic mitochondrial oxidant stress in SNc and LC neurons, and that MAO inhibition is neuroprotective [36,37,42], we hypothesized that chronic meth administration would result in axon loss first, followed by somatic loss, consistent with a dying-back pattern of neurodegeneration. To test this hypothesis, male mice were administered meth (5 mg/kg; i.p.) or saline for 14, 21, or 28 consecutive days, after which the axon lengths and numbers of SNc and LC neurons were stereologically quantified. To confirm MAO-dependence of degeneration, a separate group of mice were treated with the MAO inhibitor phenelzine (20 mg/kg; i.p.) as a 30-min pretreatment prior to each meth injection. To determine whether SNc and LC neurons in female subjects are similarly vulnerable to chronic meth-induced degeneration, female subjects were administered saline or meth (5 mg/kg; i.p.) for 28 consecutive days, and axon lengths and numbers of neurons in the SNc and LC were stereologically quantified.

## 2. Results

### 2.1. Chronic Methamphetamine Administration Resulted in Axonal Loss Prior to Somatic Loss of Substantia Nigra Pars Compacta Dopamine Neurons in Male Mice

**Figure 1:**
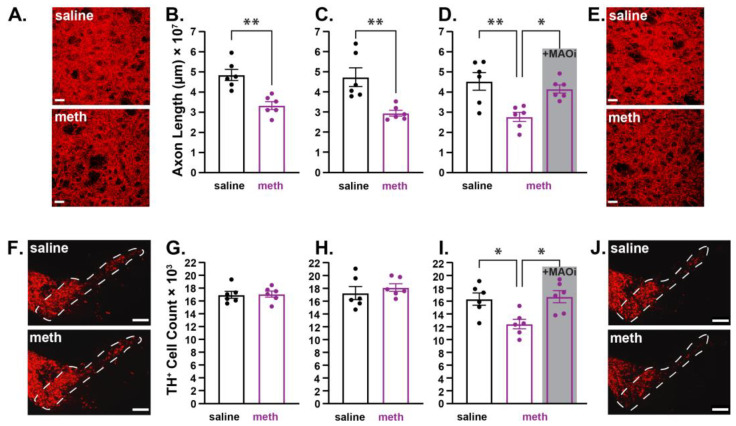
Repeated methamphetamine administration in male mice resulted in axon loss prior to somatic loss in substantia nigra pars compacta dopamine neurons. Male mice were treated with saline or methamphetamine (meth; 5 mg/kg) for 14, 21, or 28 consecutive days, after which substantia nigra pars compacta (SNc) axonal lengths in the dorsolateral striatum (DLS) and numbers of dopamine neurons in the SNc were stereologically quantified. (**A**) Representative images of SNc axons stained for tyrosine hydroxylase (TH^+^; red) in the DLS from a male mouse treated with saline (**top**) and meth (**bottom**) for 14 consecutive days; scale bars denote 20 µm. (**B**) SNc axon length decreased in mice treated with meth for 14 days (*n* = 6) compared to saline-treated control mice (*n* = 6). Data analyzed using unpaired *t*-test (*t*(10) = 4.483, *p* = 0.0012, two-tailed). (**C**) Administration of meth (5 mg/kg) for 21 days also decreased SNc axonal length (saline: *n* = 6; meth: *n* = 6; *t*(10) = 3.669, *p* = 0.0043, two-tailed). (**D**) Male mice were administered saline (*n* = 6), meth (*n* = 6) or meth with a 30-min phenelzine (20 mg/kg; +MAOi; *n* = 6) pretreatment for 28 days; 28-day meth treatment decreased SNc axonal length, and this decrease was prevented by pretreatment with phenelzine. Data analyzed using one-way ANOVA (F_(2,15)_ = 9.108, *p* = 0.0026) with Tukey’s post hoc analysis (saline vs. meth, *p* = 0.0028; saline vs. +MAOi, *p* = 0.6713; meth vs. +MAOi, *p* = 0.0158). (**E**) Representative images of SNc axons stained for tyrosine hydroxylase (TH^+^) in the DLS from a male mouse treated with saline (**top**) and meth (5 mg/kg; **bottom**) for 28 consecutive days; scale bars denote 20 µm. (**F**) Representative images of TH^+^ SNc neurons from a male mouse treated with saline (**top**) and meth (**bottom**) for 14 consecutive days; scale bars denote 200 µm. (**G**) The numbers of TH^+^ neurons in the SNc did not decrease in mice treated with meth (*n* = 6) for 14 days compared to saline-treated mice (*n* = 6). Data analyzed using unpaired *t*-test (*t*(10) = 0.1675, *p* = 0.8703, two-tailed). (**H**) Administration of meth (5 mg/kg) for 21 days also did not alter the number of TH^+^ neurons in the SNc (saline: *n* = 6; meth: *n* = 6; *t*(10) = 0.7319, *p* = 0.4810, two-tailed). (**I**) Male mice were administered saline (*n* = 6), meth (*n* = 6), or meth with a 30-min phenelzine (20 mg/kg; +MAOi; *n* = 6) pretreatment for 28 consecutive days; 28-day meth treatment decreased the number of TH^+^ SNc neurons, and this decrease was prevented by pretreatment with phenelzine. Data analyzed using one-way ANOVA (F_(2,15)_ = 7.095, *p* = 0.0068) with Tukey’s post hoc analysis (saline vs. meth, *p* = 0.0188; saline vs. +MAOi, *p* = 0.9515; meth vs. +MAOi, *p* = 0.0103). (**J**) Representative images of TH^+^ SNc neurons from a male mouse treated with saline (**top**) and meth (**bottom**) for 28 consecutive days; scale bars denote 200 µm; * *p* ≤ 0.05, ** *p* ≤ 0.01.

In ex vivo brain slices, the bath perfusion of meth (10 µM) increased MAO-dependent axonal but not somatic mitochondrial oxidant stress in SNc neurons [38]; in vivo, the chronic administration of meth (5 mg/kg; i.p.) for 28 consecutive days resulted in degeneration of SNc dopamine neurons, which is prevented by pretreating subjects with a mitochondrial antioxidant or a MAO inhibitor [36,37]; this suggests that meth-induced MAO-dependent axonal mitochondrial oxidant stress is a driver of degeneration, and that the deleterious effects of repeated meth exposure impact the axons first. To test whether axonal loss precedes somatic loss, male mice were administered saline or meth (5 mg/kg) for 14, 21, or 28 consecutive days, after which brains were collected and SNc axon lengths in the dorsolateral striatum (DLS) and numbers of dopamine neurons in the SNc were stained for TH (TH^+^). The lengths of TH^+^ axons in the DLS and numbers of TH^+^ neurons in the SNc were stereologically quantified by an experimenter blinded to the treatment histories. Fourteen days of meth administration decreased SNc axonal lengths in the DLS (Figure 1A,B), but had no effect on the numbers of TH^+^ neurons in the SNc (Figure 1F,G). Similarly, 21 days of meth (5 mg/kg) decreased SNc axonal lengths in the DLS (Figure 1C), but still had no effect on the numbers of TH^+^ SNc neurons (Figure 1H). However, consistent with prior studies [36,37], a chronic 28-day treatment of meth resulted in a decrease in both SNc axonal length (Figure 1D,E) and numbers of TH^+^ neurons in the SNc (Figure 1I,J). We have previously shown that rasagiline, a MAO-B inhibitor with FDA approval for the treatment of Parkinson’s disease, prevents chronic meth-induced SNc degeneration [36,37]. In the current study, we tested a second clinically available MAO inhibitor, phenelzine, which inhibits both MAO-A and MAO-B isoforms, and is FDA-approved for the treatment of panic disorder, social anxiety disorder, and treatment-resistant depression. Pre-treating mice with phenelzine (20 mg/kg) 30 min prior to each meth administration prevented both axonal (Figure 1D) and somatic loss of SNc dopamine neurons (Figure 1I).

### 2.2. Chronic Administration of Methamphetamine Resulted in Axonal Loss Prior to Somatic Loss of Locus Coeruleus Norepinephrine Neurons in Male Mice

**Figure 2:**
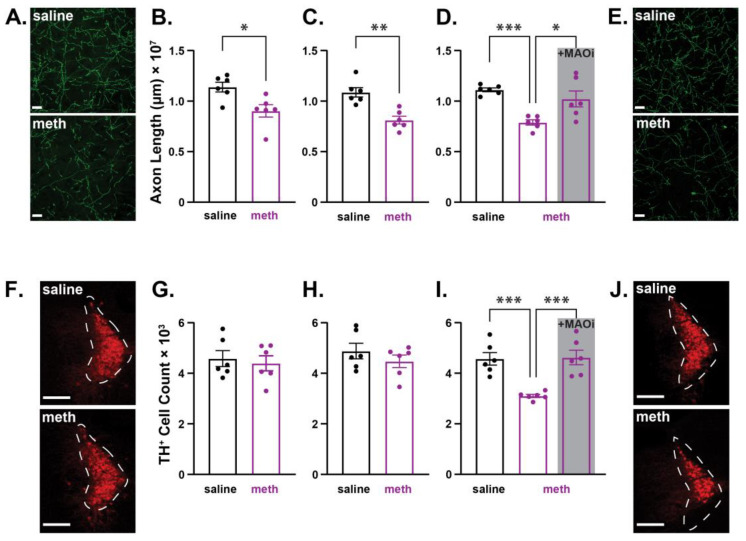
Repeated methamphetamine administration in male mice resulted in axonal loss prior to somatic loss in locus coeruleus norepinephrine neurons. Male mice were treated with saline or methamphetamine (meth; 5 mg/kg) for 14, 21, or 28 consecutive days, after which locus coeruleus (LC) axonal lengths in the M1 motor cortex and numbers of norepinephrine neurons in the LC were stereologically quantified. (**A**) Representative images of LC axons stained for the norepinephrine transporter (NET^+^; green) in the M1 motor cortex from a male mouse treated with saline (**top**) and meth (**bottom**) for 14 consecutive days; scale bars denote 20 µm. (**B**) LC axonal length decreased in mice treated with meth for 14 days (*n* = 6) compared to saline-treated control mice (*n* = 6). Data analyzed using unpaired *t*-test (*t*(10) = 3.102, *p* = 0.0131, two-tailed). (**C**) Administration of meth for 21 consecutive days also decreased LC axonal length (saline: *n* = 6; meth: *n* = 6; *t*(10) = 4.562, *p* = 0.0010, two-tailed). (**D**) Male mice were administered saline (*n* = 6), meth (*n* = 6) or meth with a 30-min phenelzine (20 mg/kg; +MAOi; *n* = 6) pretreatment for 28 consecutive days, and LC axonal lengths in the M1 motor cortex were stereologically quantified. Chronic 28-day meth treatment decreased LC axonal length compared to saline-treated controls, and this decrease was prevented by pretreatment with phenelzine. Data analyzed using one-way ANOVA (F_(2,15)_ = 11.41, *p* = 0.0010) with Tukey’s post hoc analysis (saline vs. meth, *p* = 0.0009; saline vs. +MAOi, *p* = 0.4192; meth vs. +MAOi, *p* = 0.0119). (**E**) Representative images of NET^+^ LC axons in the M1 motor cortex from a male mouse treated with saline (**top**) and meth (**bottom**) for 28 consecutive days; scale bars denote 20 µm. (**F**) Representative images of LC neurons stained for tyrosine hydroxylase (TH^+^; red) from a male mouse treated with saline (**top**) and meth (**bottom**) for 14 consecutive days; scale bars denote 200 µm. (**G**) The numbers of TH^+^ LC neurons did not decrease in mice treated with meth for 14 days (*n* = 6) compared to saline-treated control mice (*n* = 6). Data analyzed using unpaired *t*-test (*t*(10) = 0.4326, *p* = 0.6745, two-tailed). (**H**) Administration of meth for 21 consecutive days also did not decrease the number of TH^+^ LC neurons (saline: *n* = 6; meth: *n* = 6; *t*(10) = 1.026, *p* = 0.3293, two-tailed). (**I**) Repeated 28-day meth treatment decreased the number of TH^+^ LC neurons compared to saline-treated controls, and this decrease was prevented by pretreatment with phenelzine (saline: *n* = 6, meth: *n* = 6, and +MAOi *n* = 6 mice). Data analyzed using one-way ANOVA (F_(2,15)_ = 15.08, *p* = 0.0003) with Tukey’s post hoc analysis (saline vs. meth, *p* = 0.0008; saline vs. +MAOi, *p* = 0.9837; meth vs. +MAOi, *p* = 0.0006). (**J**) Representative images of TH^+^ LC neurons from a male mouse treated with saline (**top**) and meth (**bottom**) for 28 consecutive days; scale bars denote 200 µm; * *p* ≤ 0.05, ** *p* ≤ 0.01 *** *p* ≤ 0.001.

In addition to targeting dopaminergic neurons, meth also binds to and induces dysfunction of VMAT2 in LC norepinephrine neurons [41]. Consistent with meth effects in SNc dopamine neurons [36,37], meth increases MAO-dependent mitochondrial oxidant stress in LC axons but not in the soma, and chronic in vivo meth administration to male mice results in LC degeneration that is prevented by MAO inhibition [42]. To test whether meth-induced LC degeneration follows the same pattern as that observed in SNc neurons (i.e., axonal loss prior to somatic loss; Figure 1), LC axons in the M1 motor cortex were stained for the norepinephrine transporter (NET^+^), and norepinephrine neurons in the LC were stained for TH from male mice treated with saline or meth (5 mg/kg; i.p.) for 14, 21, or 28 days. Consistent with the results in SNc neurons from male mice (Figure 1), chronic meth administration resulted in LC axon loss in the M1 motor cortex prior to somatic loss (Figure 2). Repeated meth administration for 14 (Figure 2A,B) and 21 (Figure 2C) days decreased LC axonal length, but had no effect on the number of TH^+^ neurons in the LC (Figure 2F–H), whereas 28 days of administration significantly decreased both axonal length (Figure 2D,E) and the number of TH^+^ LC neurons (Figure 2I,J); 28-day meth-induced deficits in LC axon lengths and numbers of TH^+^ LC neurons were prevented by pre-treating with the MAO inhibitor phenelzine (20 mg/kg; Figure 2D,I).

### 2.3. Female Mice were Resistant to Chronic Methamphetamine-Induced Degeneration of Substantia Nigra Pars Compacta Dopamine Neurons

**Figure 3:**
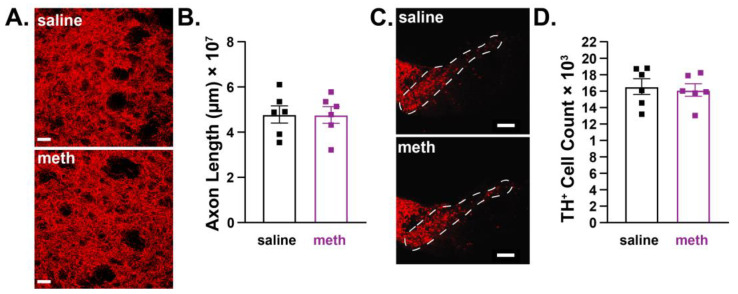
Chronic methamphetamine administration had no effect on axon length or numbers of tyrosine hydroxylase-stained substantia nigra pars compacta dopamine neurons in female mice. Female mice were treated with saline or methamphetamine (meth; 5 mg/kg) for 28 consecutive days, after which substantia nigra pars compacta (SNc) axonal length in the dorsolateral striatum (DLS) and numbers of dopamine neurons in the SNc were stereologically quantified. (**A**) Representative images of SNc axons stained for tyrosine hydroxylase (TH^+^; red) in the DLS from a female mouse treated with saline (**top**) and meth (**bottom**) for 28 consecutive days; scale bars denote 20 µm. (**B**) SNc axonal length was unchanged by 14 days of meth (*n* = 6) compared to saline-treated mice (*n* = 6). Data analyzed using unpaired *t*-test (*t*(10) = 0.03984, *p* = 0.9690, two-tailed). (**C**) Representative images of SNc neurons stained for tyrosine hydroxylase (TH^+^) from a female mouse treated with saline (**top**) and meth (**bottom**) for 28 consecutive days; scale bars denote 200 µm. (**D**) The numbers of TH^+^ SNc dopamine neurons in female mice were unchanged by chronic meth administration (saline: *n* = 6 mice; meth: *n* = 6 mice). Data analyzed using unpaired *t*-test (*t*(10) = 0.3416, *p* = 0.7397, two-tailed).

Acute binge models wherein subjects are repeatedly administered meth over the course of one day or given a single high dose consistently show deleterious effects on SNc axons in male subjects [14,17,21,22,25,27], whereas female subjects are resistant to the deleterious effects of an acute meth binge [43,44,45,46,47,48,49,50]. To determine whether SNc neurons in female subjects are similarly resistant to the effects of chronic meth, female mice were administered saline or meth (5 mg/kg) for 28 consecutive days followed by stereological analysis of SNc axonal lengths in the DLS and numbers of TH^+^ neurons in the SNc. Congruent with results from studies using acute binge models [43,44,45,46,47,48,49,50], female mice were resistant to chronic meth-induced degeneration of SNc neurons. There was no difference in the length of SNc axons in the DLS (Figure 3A,B) or the number of TH^+^ neurons in the SNc (Figure 3C,D) between mice administered saline or meth for 28 consecutive days.

### 2.4. Female Mice were Resistant to Chronic Methamphetamine-Induced Degeneration of Locus Coeruleus Norepinephrine Neurons

**Figure 4:**
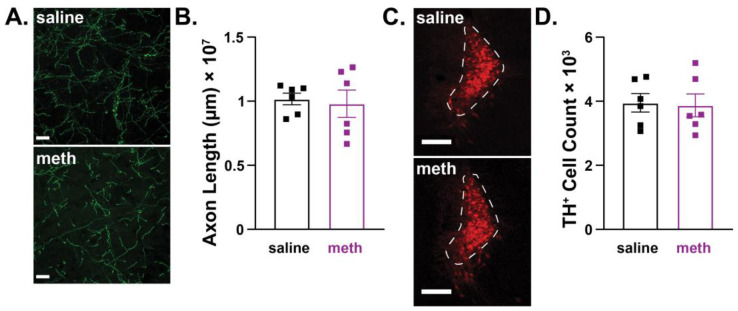
Chronic methamphetamine administration had no effect on axon lengths or numbers of locus coeruleus norepinephrine neurons in female mice. Female mice were treated with saline or methamphetamine (meth; 5 mg/kg) for 28 consecutive days, after which locus coeruleus (LC) axonal lengths in the M1 motor cortex and numbers of norepinephrine neurons in the LC were stereologically quantified. (**A**) Representative images of LC axons stained for the norepinephrine transporter (NET^+^; green) in the M1 motor cortex from a female mouse treated with saline (**top**) and meth (**bottom**) for 28 days; scale bars denote 20 µm. (**B**) NET^+^ LC axonal length was unchanged by 28-day meth (*n* = 6) compared to saline-treated control mice (*n* = 6). Data analyzed using unpaired *t*-test (*t*(10) = 0.3210, *p* = 0.7548, two-tailed). (**C**) Representative images of LC neurons stained for tyrosine hydroxylase (TH^+^; red) from a female mouse treated with saline (**top**) and meth (**bottom**) for 28 consecutive days; scale bars denote 200 µm. (**D**) The numbers of TH^+^ LC neurons were unchanged by chronic 28-day treatment of meth (*n* = 6) compared to saline-treated control mice (*n* = 6). Data analyzed using unpaired *t*-test (*t*(10) = 0.1698, *p* = 0.8692, two-tailed).

To determine whether LC neurons were resistant to chronic meth-induced degeneration in female mice, the lengths of NET^+^ LC axons in the M1 motor cortex and numbers of TH^+^ cells in the LC were quantified after chronic 28-day administration of saline or meth (5 mg/kg). Consistent with results in SNc dopamine neurons (Figure 3), LC norepinephrine neurons in female mice were resistant to chronic meth-induced degeneration. There were no differences in the lengths of NET^+^ axons in the M1 motor cortex (Figure 4A,B) or numbers of TH^+^ neurons in the LC between female subjects treated with saline or meth (Figure 4C,D).

## 3. Discussion

We recently reported that chronic 28-day in vivo administration of meth (5 mg/kg) results in a loss of SNc dopamine and LC norepinephrine neurons in male mice [36,37,42]. Results from the current study extend these findings to show that in both SNc dopamine and LC norepinephrine neurons, meth administration produces axonal deficits prior to somatic loss, suggesting that chronic meth administration produces a dying-back pattern of degeneration in both SNc and LC neurons. The observed losses of TH^+^ neurons in the SNc and LC are unlikely to be the result of phenotypic suppression, as our prior studies have shown decreased numbers of NeuN-, the neuronal-specific nuclear splicing regulator Fox 3 [51] stained cells in the SNc and LC [36,42], supporting the interpretation of overt cell loss. Furthermore, the number of SNc neurons and optical density of nigrostriatal axons fluorescently labelled by genetically encoded TdTomato were also decreased by chronic 28-day meth [37]. However, it is possible that phenotypic suppression occurs prior to cell loss, as recently reported in a mouse model of Parkinson’s disease [52]. Our prior studies also demonstrated the neuroprotective efficacy of rasagiline and isradipine [36,37,42]. Rasagiline is an irreversible MAO-B inhibitor that is FDA-approved for treatment of Parkinson’s disease, and isradipine is an L-type calcium channel inhibitor that is an FDA-approved dihydropyridine antihypertensive medication. Current results expand the list of clinically available medications that attenuate meth-induced degeneration to include phenelzine, an irreversible MAO-A/B inhibitor FDA-approved for treatment-resistant depression, panic disorder, and social anxiety disorder. MAO inhibition using the MAO-A selective inhibitor clorgyline also attenuates meth-induced mitochondrial oxidant stress in SNc axons [38]; future studies will be necessary to determine whether MAO-A inhibition is similarly effective at preventing meth-induced SNc and/or LC degeneration. A third key outcome from our investigation is a robust sex difference wherein female subjects were resistant to chronic meth-induced degeneration.

Chronic 28-day meth administration to male mice resulted in a ~30% decrease in the numbers of TH^+^ neurons in the SNc and LC as well as in corresponding axon lengths in the DLS and M1 motor cortex, a magnitude of effect that is consistent with our prior studies [36,42]. However, the same treatment paradigm had no effect on the axon length or number of SNc or LC neurons in female mice. Female subjects have similarly been shown to be resistant to the neurotoxicity that results from an acute meth binge [43,44,45,46,47,48,49,50]. The mechanism underlying these observed sex differences regarding meth neurotoxicity is unclear. One potential mechanism may be linked to L-type calcium channels. Cav_1.3_ L-type calcium channel activity contributes to mitochondrial oxidant stress in SNc and LC neurons [53,54,55,56,57]; inhibition of L-type calcium channels with isradipine attenuates mitochondrial oxidant stress in SNc and LC neurons, is neuroprotective in mouse models of Parkinson’s disease, and prevents chronic meth-induced degeneration of SNc and LC neurons in male mice [36,42,54,56,58,59]. In cultured striatal neurons, 17β-estradiol inhibits L-type calcium channel-mediated currents, and the magnitude of this effect is larger in neurons from female subjects [60]. Therefore, we would predict that mitochondrial oxidant stress in SNc and LC neurons from female subjects would be less than that in males due to inhibition of L-type calcium channels by endogenous 17β-estradiol, and that this endogenous mechanism of L-type calcium channel inhibition could potentially account for the observed sex difference. Future studies are needed to test this hypothesis and determine the impact of sex hormones on mitochondrial oxidant stress and its implications for neurodegeneration. Whether the observed resistance to meth-induced degeneration in females is long-lasting is also in need of further study. In male rats trained to self-administer meth for 14 days, evidence of nigrostriatal axon loss did not become apparent until 14 days of abstinence, with evidence of SNc degeneration appearing at 28 days of abstinence [61]. This suggests that degenerative processes may continue to evolve throughout periods of abstinence. Further investigation is needed to fully examine and explore mechanisms of potential degeneration during abstinence, and whether resistance to degeneration in female subjects persists. Overall, the neurotoxic effects of meth on catecholaminergic systems and the possibility of continued degeneration during abstinence raise significant concerns regarding the potential impact of meth abuse on neurodegenerative diseases.

Parkinson’s disease (PD) is the most common neurodegenerative movement disorder, and the second-most common neurodegenerative disease overall. The deleterious effects of meth are strikingly similar to the neurodegeneration seen in PD. In PD, both SNc and LC neurons are particularly vulnerable to degeneration [62,63], a feature recapitulated by chronic meth administration [36,42]. Neurodegeneration in PD has been hypothesized to advance in a dying-back pattern wherein nigrostriatal axons are lost, followed by overt SNc degeneration [64,65,66] in a manner that is analogous to the observed effects of chronic meth on both SNc and LC neurons in male mice. Indeed, clinical research has demonstrated that at PD onset, patients display more severe markers of striatal axon loss than SNc cell loss, with axonal loss progressing faster than cell loss in the following 10 years [67]. Like in SNc neurons, LC axon loss also appears to occur prior to somatic loss of LC norepinephrine neurons in PD [68,69]. Meth treatment in rodents has also been shown to increase the expression of α-synuclein, a hallmark of PD, in the nigrostriatal system and gut [70,71,72]. Another parallel between meth-induced neurotoxicity and PD is the presence of a sex difference, with female subjects displaying relative resistance in both cases. In humans, the incidence of idiopathic PD is approximately 1.5x more common in men than women, and has an earlier onset [73]. Given the similarities between meth neurotoxicity and PD, it is perhaps not surprising that meth abuse is associated with an increased the risk for developing PD [74,75,76,77,78,79], although see [80,81]. Whether disease trajectory and severity are altered in PD patients with a history of meth abuse is unclear; further research is required to determine longitudinal effects, and to explore potential converging or overlapping mechanisms driving this degeneration. In addition to PD, our findings, past and present, on meth-induced neurodegeneration share connections with another neurodegenerative disorder: Alzheimer’s disease (AD).

AD is the most common neurodegenerative disease and most common form of dementia, accounting for between 50–75% of dementia cases worldwide [82,83]. In AD, hippocampal and cortical degeneration is quite prominent; however, monoaminergic neurons, including LC neurons, are also vulnerable to degeneration. Clinical studies report an approximate 38–88% loss of TH^+^, neuromelanin-, and dopamine β-hydroxylase-labeled LC neurons in post-mortem tissue [84,85,86]. Clinical studies also report that AD patients have decreased LC volume [87] and decreased LC signal intensity in neuromelanin-sensitive MRI scans [88,89]. Importantly, decreases in TH^+^ LC neurons are associated with worsened cognitive function and increased AD neuropathology in human subjects [84]. Preclinical AD rodent models also show LC degeneration. Aged APP/PS1 mice have fewer TH^+^ and NET^+^ LC neurons than age-matched controls [90,91,92,93]. This loss of TH^+^ LC neurons is also observed in the Tau P301S/DBH mouse model [94]. The presence of AD-related pathology in the LC and LC neuron loss may even occur during pre-symptomatic and mild cognitive impairment stages of AD prior to glutamatergic degeneration [95,96]. LC degeneration and loss of noradrenergic signaling is particularly concerning in AD, as it may contribute to disease progression and pathogenesis; pre-clinical studies show that noradrenergic signaling attenuates amyloid-β deposition, and lesioning the LC increases amyloid-β pathology and neuroinflammation [97,98,99,100,101]. Additionally, LC lesions in a mouse tauopathy model of AD increases hippocampal degeneration, inflammation, and mortality [97]. Therefore, the LC appears to play a protective role in AD. Although there is a paucity of studies examining the relationship between meth and AD in vivo, 15 mg/kg (i.p.) of meth injected every 12 h for 8 weeks increases amyloid-β protein and amyloid precursor protein levels in the hippocampus of non-transgenic C57Bl/6J mice [102]. Additionally, meth exposure in vitro has also shown increased amyloid-β and hyperphosphorylated tau pathology [102,103,104]. While epidemiological evidence linking meth and AD is lacking, the neurotoxic effect of chronic meth on LC neurons shown in the current report and prior study [42] suggest that a history of meth abuse could be a potential risk factor for AD, and is in need of further investigation.

## 4. Conclusions

We recently reported that chronic 28-day administration of meth results in axonal and somatic degeneration of SNc dopamine and LC norepinephrine neurons in male mice [36,37,42]. These deleterious effects of meth were further shown to be prevented by administration of the clinically available MAO-B inhibitor rasagiline and L-type calcium channel inhibitor isradipine. Results from the current report expand upon our prior studies to show that in male mice, axon loss precedes somatic loss in both SNc and LC neurons with 14- and 21-day administration of meth, decreasing axonal length without altering the number of SNc or LC neurons; meanwhile, consistent with our prior investigations [36,37,42], 28 days of meth resulted in both axonal and somatic loss. Similar to the neuroprotective effect of the MAO-B inhibitor rasagiline [36,37,42], phenelzine, a non-specific MAO-A/B inhibitor that is also clinically available, prevented meth-induced neurodegeneration. In stark contrast to results in male mice, we found that female mice were resistant to meth-induced SNc and LC degeneration. The pattern of degeneration observed in male mice and the sex difference parallels that seen in Parkinson’s disease, the most common neurodegenerative movement disorder, and patients with a history of meth abuse are reported to have an increased risk for developing Parkinson’s disease [74,75,79]. Whether the mechanisms underlying degeneration are shared between meth and Parkinson’s disease requires further investigation. In addition to concerns for Parkinson’s disease, we believe there may also be increased risk for Alzheimer’s disease, the most common neurodegenerative disease. LC neurons degenerate in Alzheimer’s disease, and LC degeneration has been linked to pathogeneses related to Alzheimer’s [105]. Taken together, the deleterious effects of meth abuse may extend beyond neurotoxicity, and perhaps set the stage for the development of Parkinson’s and/or Alzheimer’s disease. Based on the current report, it would seem that this potential risk is relegated to male subjects, given that female mice were resistant to meth induced neurodegeneration; however, further study is needed to determine whether the observed resistance in females persists, or if perhaps the deleterious effects of meth simply take longer to manifest.

## 5. Materials and Methods

### 5.1. Experimental Subjects

Male and female C57Bl/6J mice were bred in-house. All subjects were group housed, maintained on a 12-h light/dark cycle, and provided free access to food and water in the home cage throughout the study. The procedures were reviewed and approved by the University of Minnesota Animal Care and use Committee, and conform to the National Institutes of Health Guide for the Care and Use of Laboratory Animals.

### 5.2. In Vivo Drug Administration

The male and female mice began in vivo treatments at approximately 8 weeks of age. The subjects were administered saline (10 mL/kg; General Laboratory Products) or (+)-methamphetamine hydrochloride (meth, 5 mg/kg; Sigma-Aldrich, St. Louis, MO, USA) for 14, 21, or 28 consecutive days in the home cage. To test whether monoamine oxidase (MAO) inhibition prevents degeneration, the irreversible MAO-A/B inhibitor phenelzine (20 mg/kg; Sigma-Aldrich) was administered as a 30-min pretreatment prior to each meth injection. Meth and phenelzine were dissolved in sterile saline, and all of the injections were intraperitoneal. The dose of meth and 28-day duration were based on our prior studies demonstrating meth-induced degeneration of SNc and LC neurons in male mice [36,37,42]. The dose of phenelzine (20 mg/kg) was chosen based on it being a behaviorally relevant dose in mice [106].

### 5.3. Immunohistochemistry

The tissue collection, sectioning, processing, and staining procedures were consistent with our prior studies [36,42]. The mice were euthanized within 12 h of the last treatment via terminal anesthesia using ketamine (50 mg/kg)/xylazine (4.5 mg/kg) followed by transcardial perfusion with 4% paraformaldehyde in phosphate-buffered saline (PBS). The brains were extracted, post-fixed overnight in 4% paraformaldehyde in PBS, and cryoprotected in 30% sucrose in PBS. The fixed brains were sectioned (40 µm) using a microtome (Leica SM2010R, Deerfield, IL, USA), and sections spanning the dorsolateral striatum (DLS), anterior portion of the primary motor cortex (M1), substantia nigra pars compacta (SNc), and locus coeruleus (LC) were collected. Every third brain section spanning the SNc and LC was stained for tyrosine hydroxylase (TH^+^), resulting in 11–13 SNc and 7–8 LC sections. Antibodies were tested for non-specific staining in-house by incubating the tissue with either primary or secondary antibody omission; fluorescent staining was not observed after primary or secondary antibody omission. Additionally, as per manufacturer websites, rabbit anti-TH polyclonal primary antibody (AB152, Millipore, Burlington, MA, USA) was tested in the corpus striatum, sympathetic nerve terminal, and adrenal gland tissue as positive controls, with liver tissue as a negative control, and Western blot analysis; mouse IgG1 anti-NET primary antibody (MA5-24647, ThermoFisher, Waltham, MA, USA) was tested in rat locus coeruleus, mouse cortex and hippocampus, and human prostate, placenta, and locus coeruleus tissue as positive controls. Secondary antibodies (Alexa 555 donkey anti-rabbit (A-31572, Invitrogen, Waltham, MA, USA) and Alexa 488 donkey anti-mouse (A-21202, Invitrogen)) have been evaluated by the manufacturer for non-specific staining in cell cultures using primary antibody exclusion and isotype controls. For the striatum and primary motor cortex, every sixth section was stained for TH^+^ and the norepinephrine transporter (NET^+^), respectively, resulting in 4 sections for each region. Prior to immunostaining, the sections were first treated with 20% formic acid for antigen retrieval, followed by blocking with 5% normal donkey serum. TH^+^ staining of the SNc, LC, and DLS sections consisted of incubation with the primary antibody (rabbit anti-TH polyclonal AB152, Millipore 1:2000), followed by washing and incubation with Alexa 555 donkey anti-rabbit secondary antibody (A-31572, Invitrogen, 1:200). NET^+^ staining of LC axons in the primary motor cortex were carried out using mouse IgG1 anti-NET primary antibody (MA5-24647, ThermoFisher; 1:1000) and Alexa 488 donkey anti-mouse (A-21202, Invitrogen) secondary antibody (1:200). The stained sections were mounted on glass slides (Electron Microscopy Sciences, Hatfield, PA, USA) with ProLong Diamond Antifade Mountant (Invitrogen), then coverslipped and stored at −20 °C.

### 5.4. Stereological Quantification

The stained sections were analyzed using a Zeiss microscope with a motorized stage and digital camera controlled by StereoInvestigator software version 2020 (MBF Bioscience, Williston, VT, USA). Anatomical boundaries were delineated using a 2.5X/0.085NA objective lens and stereological counting using the optical fractionator and Spaceballs probes performed using a 63×/1.4NA lens. Using the optical fractionator probe, cells were individually marked within counting frames, and total numbers of cells were calculated with StereoInvestigator software. The Spaceballs probe uses a virtual hemisphere superimposed over tissue through the *z*-plane; the axons were marked where they crossed the hemispheres at counting sites, and total axon lengths were calculated using StereoInvestigator software. For further reading on the optical fractionator and Spaceballs probes, see [107,108]. The TH^+^ cells were counted throughout the SNc with a 150 μm × 150 μm counting frame and grid size of 250 μm × 275 μm [36]. A 150 µm × 150 µm counting frame and 275 µm × 175 µm grid size was used to count the LC neurons [42]. Somatic counting in the SNc and LC was performed using the optical fractionator probe with 3 µm guard zones; the stereological parameters used resulted in a Gunderson coefficient of error (m = 1) of 0.03 (SNc TH^+^ neurons) and 0.05 (LC TH^+^ neurons) or less. The SNc axons in the dorsolateral striatum (DLS) and LC axons in the primary motor cortex were quantified using the Spaceballs probe with a hemisphere of 7 μm and 20 μm radius, respectively. For SNc axons in the DLS a grid size of 275 μm × 275 μm, was used [36], and for LC axons in the primary motor cortex a grid size of 250 µm × 250 µm was used [42]. Quantification of SNc axons in the DLS and LC axons in the primary motor cortex was carried out using a guard zone of 3 μm; the described parameters resulted in a Gundersen coefficient of error (m = 1) of 0.08 (DLS TH^+^ axons) and 0.06 (motor cortex NET^+^ axons) or less. All stereological counting was performed by an experimenter blinded to the treatment conditions.

### 5.5. Statistical Analysis

All datasets passed Shapiro–Wilk normality testing, and were analyzed using unpaired Student *t*-tests or one-way ANOVAs with Tukey’s post hoc analysis; α = 0.05. The statistical analyses were performed using GraphPad Prism Software, and data are presented as histograms depicting mean and standard error of the mean overlayed with individual dot plots. Detailed statistical reporting for all of the experiments is provided in the figure legends.

## Data Availability

Data will be made available upon reasonable request.

## References

[1] Substance Abuse and Mental Health Services Administration (2017). Key Substance Use and Mental Health Indicators in the United States: Results from the 2016 National Survey on Drug Use and Health.

[2] Substance Abuse and Mental Health Services Administration (2022). Key Substance Use and Mental Health Indicators in the United States: Results from the 2021 National Survey on Drug Use and Health.

[3] Jones C.M., Houry D., Han B., Baldwin G., Vivolo-Kantor A., Compton W.M. (2022). Methamphetamine use in the United States: Epidemiological update and implications for prevention, treatment, and harm reduction. Ann. N. Y. Acad. Sci..

[4] Jayanthi S., Daiwile A.P., Cadet J.L. (2021). Neurotoxicity of methamphetamine: Main effects and mechanisms. Exp. Neurol..

[5] Shaerzadeh F., Streit W.J., Heysieattalab S., Khoshbouei H. (2018). Methamphetamine neurotoxicity, microglia, and neuroinflammation. J. Neuroinflam..

[6] McCann U.D., Wong D.F., Yokoi F., Villemagne V., Dannals R.F., Ricaurte G.A. (1998). Reduced striatal dopamine transporter density in abstinent methamphetamine and methcathinone users: Evidence from positron emission tomography studies with [^11^C]WIN-35,428. J. Neurosci..

[7] Volkow N.D., Chang L., Wang G.J., Fowler J.S., Leonido-Yee M., Franceschi D., Sedler M.J., Gatley S.J., Hitzemann R., Ding Y.S. (2001). Association of dopamine transporter reduction with psychomotor impairment in methamphetamine abusers. Am. J. Psychiatry.

[8] McCann U.D., Kuwabara H., Kumar A., Palermo M., Abbey R., Brasic J., Ye W., Alexander M., Dannals R.F., Wong D.F. (2008). Persistent cognitive and dopamine transporter deficits in abstinent methamphetamine users. Synapse.

[9] Johanson C.E., Frey K.A., Lundahl L.H., Keenan P., Lockhart N., Roll J., Galloway G.P., Koeppe R.A., Kilbourn M.R., Robbins T. (2006). Cognitive function and nigrostriatal markers in abstinent methamphetamine abusers. Psychopharmacology.

[10] Sekine Y., Iyo M., Ouchi Y., Matsunaga T., Tsukada H., Okada H., Yoshikawa E., Futatsubashi M., Takei N., Mori N. (2001). Methamphetamine-related psychiatric symptoms and reduced brain dopamine transporters studied with PET. Am. J. Psychiatry.

[11] Moszczynska A., Callan S.P. (2017). Molecular, Behavioral, and Physiological Consequences of Methamphetamine Neurotoxicity: Implications for Treatment. J. Pharmacol. Exp. Ther..

[12] Wilson J.M., Kalasinsky K.S., Levey A.I., Bergeron C., Reiber G., Anthony R.M., Schmunk G.A., Shannak K., Haycock J.W., Kish S.J. (1996). Striatal dopamine nerve terminal markers in human, chronic methamphetamine users. Nat. Med..

[13] Kitamura O., Tokunaga I., Gotohda T., Kubo S. (2007). Immunohistochemical investigation of dopaminergic terminal markers and caspase-3 activation in the striatum of human methamphetamine users. Int. J. Leg. Med..

[14] Ares-Santos S., Granado N., Espadas I., Martinez-Murillo R., Moratalla R. (2014). Methamphetamine causes degeneration of dopamine cell bodies and terminals of the nigrostriatal pathway evidenced by silver staining. Neuropsychopharmacology.

[15] Blaker A.L., Rodriguez E.A., Yamamoto B.K. (2019). Neurotoxicity to dopamine neurons after the serial exposure to alcohol and methamphetamine: Protection by COX-2 antagonism. Brain Behav. Immun..

[16] Dang D.K., Shin E.J., Nam Y., Ryoo S., Jeong J.H., Jang C.G., Nabeshima T., Hong J.S., Kim H.C. (2016). Apocynin prevents mitochondrial burdens, microglial activation, and pro-apoptosis induced by a toxic dose of methamphetamine in the striatum of mice via inhibition of p47phox activation by ERK. J. Neuroinflam..

[17] Eyerman D.J., Yamamoto B.K. (2005). Lobeline attenuates methamphetamine-induced changes in vesicular monoamine transporter 2 immunoreactivity and monoamine depletions in the striatum. J. Pharmacol. Exp. Ther..

[18] Eyerman D.J., Yamamoto B.K. (2007). A rapid oxidation and persistent decrease in the vesicular monoamine transporter 2 after methamphetamine. J. Neurochem..

[19] Frey K., Kilbourn M., Robinson T. (1997). Reduced striatal vesicular monoamine transporters after neurotoxic but not after behaviorally-sensitizing doses of methamphetamine. Eur. J. Pharmacol..

[20] Fumagalli F., Gainetdinov R.R., Valenzano K.J., Caron M.G. (1998). Role of dopamine transporter in methamphetamine-induced neurotoxicity: Evidence from mice lacking the transporter. J. Neurosci..

[21] Granado N., Ares-Santos S., O’Shea E., Vicario-Abejón C., Colado M.I., Moratalla R. (2010). Selective vulnerability in striosomes and in the nigrostriatal dopaminergic pathway after methamphetamine administration: Early loss of TH in striosomes after methamphetamine. Neurotox. Res..

[22] Granado N., Ares-Santos S., Tizabi Y., Moratalla R. (2018). Striatal Reinnervation Process after Acute Methamphetamine-Induced Dopaminergic Degeneration in Mice. Neurotox. Res..

[23] Guillot T.S., Shepherd K.R., Richardson J.R., Wang M.Z., Li Y., Emson P.C., Miller G.W. (2008). Reduced vesicular storage of dopamine exacerbates methamphetamine-induced neurodegeneration and astrogliosis. J. Neurochem..

[24] Hogan K.A., Staal R.G., Sonsalla P.K. (2000). Analysis of VMAT2 binding after methamphetamine or MPTP treatment: Disparity between homogenates and vesicle preparations. J. Neurochem..

[25] Lohr K.M., Stout K.A., Dunn A.R., Wang M., Salahpour A., Guillot T.S., Miller G.W. (2015). Increased Vesicular Monoamine Transporter 2 (VMAT2; Slc18a2) Protects against Methamphetamine Toxicity. ACS Chem. Neurosci..

[26] Mark K.A., Soghomonian J.J., Yamamoto B.K. (2004). High-dose methamphetamine acutely activates the striatonigral pathway to increase striatal glutamate and mediate long-term dopamine toxicity. J. Neurosci..

[27] McConnell S.E., O’Banion M.K., Cory-Slechta D.A., Olschowka J.A., Opanashuk L.A. (2015). Characterization of binge-dosed methamphetamine-induced neurotoxicity and neuroinflammation. Neurotoxicology.

[28] Moszczynska A., Yamamoto B.K. (2011). Methamphetamine oxidatively damages parkin and decreases the activity of 26S proteasome in vivo. J. Neurochem..

[29] Northrop N.A., Yamamoto B.K. (2013). Cyclooxygenase activity contributes to the monoaminergic damage caused by serial exposure to stress and methamphetamine. Neuropharmacology.

[30] O’Callaghan J.P., Miller D.B. (1994). Neurotoxicity profiles of substituted amphetamines in the C57BL/6J mouse. J. Pharmacol. Exp. Ther..

[31] Reichel C.M., Ramsey L.A., Schwendt M., McGinty J.F., See R.E. (2012). Methamphetamine-induced changes in the object recognition memory circuit. Neuropharmacology.

[32] Salvatore M.F., Nejtek V.A., Khoshbouei H. (2018). Prolonged increase in ser31 tyrosine hydroxylase phosphorylation in substantia nigra following cessation of chronic methamphetamine. Neurotoxicology.

[33] Seiden L.S., Commins D.L., Vosmer G., Axt K., Marek G. (1988). Neurotoxicity in dopamine and 5-hydroxytryptamine terminal fields: A regional analysis in nigrostriatal and mesolimbic projections. Ann. N. Y. Acad. Sci..

[34] Sonsalla P.K., Jochnowitz N.D., Zeevalk G.D., Oostveen J.A., Hall E.D. (1996). Treatment of mice with methamphetamine produces cell loss in the substantia nigra. Brain Res..

[35] Truong J.G., Wilkins D.G., Baudys J., Crouch D.J., Johnson-Davis K.L., Gibb J.W., Hanson G.R., Fleckenstein A.E. (2005). Age-dependent methamphetamine-induced alterations in vesicular monoamine transporter-2 function: Implications for neurotoxicity. J. Pharmacol. Exp. Ther..

[36] Du Y., Lee Y.B., Graves S.M. (2021). Chronic methamphetamine-induced neurodegeneration: Differential vulnerability of ventral tegmental area and substantia nigra pars compacta dopamine neurons. Neuropharmacology.

[37] Graves S.M., Schwarzschild S.E., Tai R.A., Chen Y., Surmeier D.J. (2021). Mitochondrial oxidant stress mediates methamphetamine neurotoxicity in substantia nigra dopaminergic neurons. Neurobiol. Dis..

[38] Graves S.M., Xie Z., Stout K.A., Zampese E., Burbulla L.F., Shih J.C., Kondapalli J., Patriarchi T., Tian L., Brichta L. (2020). Dopamine metabolism by a monoamine oxidase mitochondrial shuttle activates the electron transport chain. Nat. Neurosci..

[39] Sulzer D., Sonders M.S., Poulsen N.W., Galli A. (2005). Mechanisms of neurotransmitter release by amphetamines: A review. Prog. Neurobiol..

[40] Freyberg Z., Sonders M.S., Aguilar J.I., Hiranita T., Karam C.S., Flores J., Pizzo A.B., Zhang Y., Farino Z.J., Chen A. (2016). Mechanisms of amphetamine action illuminated through optical monitoring of dopamine synaptic vesicles in Drosophila brain. Nat. Commun..

[41] Rothman R.B., Baumann M.H., Dersch C.M., Romero D.V., Rice K.C., Carroll F.I., Partilla J.S. (2001). Amphetamine-type central nervous system stimulants release norepinephrine more potently than they release dopamine and serotonin. Synapse.

[42] Du Y., Choi S., Pilski A., Graves S.M. (2022). Differential vulnerability of locus coeruleus and dorsal raphe neurons to chronic methamphetamine-induced degeneration. Front. Cell Neurosci..

[43] Bourque M., Dluzen D.E., Di Paolo T. (2011). Male/Female differences in neuroprotection and neuromodulation of brain dopamine. Front. Endocrinol..

[44] Bourque M., Liu B., Dluzen D.E., Di Paolo T. (2011). Sex differences in methamphetamine toxicity in mice: Effect on brain dopamine signaling pathways. Psychoneuroendocrinology.

[45] Liu B., Dluzen D.E. (2006). Effect of estrogen upon methamphetamine-induced neurotoxicity within the impaired nigrostriatal dopaminergic system. Synapse.

[46] Dluzen D.E., Mcdermott J.L. (2006). Estrogen, Testosterone, and Methamphetamine Toxicity. Ann. N. Y. Acad. Sci..

[47] Dluzen D.E., McDermott J.L. (2000). Neuroprotective role of estrogen upon methamphetamine and related neurotoxins within the nigrostriatal dopaminergic system. Ann. N. Y. Acad. Sci..

[48] Gajjar T.M., Anderson L.I., Dluzen D.E. (2003). Acute effects of estrogen upon methamphetamine induced neurotoxicity of the nigrostriatal dopaminergic system. J. Neural Transm..

[49] Dluzen D.E., Anderson L.I., Pilati C.F. (2002). Methamphetamine–gonadal steroid hormonal interactions:: Effects upon acute toxicity and striatal dopamine concentrations. Neurotoxicol. Teratol..

[50] Liu B., Dluzen D.E. (2006). Effects of Estrogen and Related Agents upon Methamphetamine-Induced Neurotoxicity within an Impaired Nigrostriatal Dopaminergic System of Ovariectomized Mice. Neuroendocrinology.

[51] Kim K.K., Adelstein R.S., Kawamoto S. (2009). Identification of neuronal nuclei (NeuN) as Fox-3, a new member of the Fox-1 gene family of splicing factors. J. Biol. Chem..

[52] González-Rodríguez P., Zampese E., Stout K.A., Guzman J.N., Ilijic E., Yang B., Tkatch T., Stavarache M.A., Wokosin D.L., Gao L. (2021). Disruption of mitochondrial complex I induces progressive parkinsonism. Nature.

[53] Guzman J.N., Sánchez-Padilla J., Chan C.S., Surmeier D.J. (2009). Robust pacemaking in substantia nigra dopaminergic neurons. J. Neurosci..

[54] Guzman J.N., Sanchez-Padilla J., Wokosin D., Kondapalli J., Ilijic E., Schumacker P.T., Surmeier D.J. (2010). Oxidant stress evoked by pacemaking in dopaminergic neurons is attenuated by DJ-1. Nature.

[55] Matschke L.A., Bertoune M., Roeper J., Snutch T.P., Oertel W.H., Rinné S., Decher N. (2015). A concerted action of L- and T-type Ca^2+^ channels regulates locus coeruleus pacemaking. Mol. Cell Neurosci..

[56] Sanchez-Padilla J., Guzman J.N., Ilijic E., Kondapalli J., Galtieri D.J., Yang B., Schieber S., Oertel W., Wokosin D., Schumacker P.T. (2014). Mitochondrial oxidant stress in locus coeruleus is regulated by activity and nitric oxide synthase. Nat. Neurosci..

[57] Zampese E., Surmeier D.J. (2020). Calcium, Bioenergetics, and Parkinson’s Disease. Cells.

[58] Chan C.S., Guzman J.N., Ilijic E., Mercer J.N., Rick C., Tkatch T., Meredith G.E., Surmeier D.J. (2007). ‘Rejuvenation’ protects neurons in mouse models of Parkinson’s disease. Nature.

[59] Ilijic E., Guzman J.N., Surmeier D.J. (2011). The L-type channel antagonist isradipine is neuroprotective in a mouse model of Parkinson’s disease. Neurobiol. Dis..

[60] Mermelstein P., Becker J., Surmeier D. (1996). Estradiol reduces calcium currents in rat neostriatal neurons via a membrane receptor. J. Neurosci..

[61] Kousik S.M., Carvey P.M., Napier T.C. (2014). Methamphetamine self-administration results in persistent dopaminergic pathology: Implications for Parkinson’s disease risk and reward-seeking. Eur. J. Neurosci..

[62] Sulzer D., Surmeier D.J. (2013). Neuronal vulnerability, pathogenesis, and Parkinson’s disease. Mov. Disord..

[63] Surmeier D.J., Obeso J.A., Halliday G.M. (2017). Selective neuronal vulnerability in Parkinson disease. Nat. Rev. Neurosci..

[64] O’Keeffe G.W., Sullivan A.M. (2018). Evidence for dopaminergic axonal degeneration as an early pathological process in Parkinson’s disease. Park. Relat. Disord..

[65] Tagliaferro P., Burke R.E. (2016). Retrograde Axonal Degeneration in Parkinson Disease. J. Park. Dis..

[66] Kurowska Z., Kordower J.H., Stoessl A.J., Burke R.E., Brundin P., Yue Z., Brady S.T., Milbrandt J., Trapp B.D., Sherer T.B. (2016). Is Axonal Degeneration a Key Early Event in Parkinson’s Disease?. J. Park. Dis..

[67] Cheng H.C., Ulane C.M., Burke R.E. (2010). Clinical progression in Parkinson disease and the neurobiology of axons. Ann. Neurol..

[68] Baloyannis S.J., Costa V., Baloyannis I.S. (2006). Morphological alterations of the synapses in the locus coeruleus in Parkinson’s disease. J. Neurol. Sci..

[69] Doppler C.E.J., Kinnerup M.B., Brune C., Farrher E., Betts M., Fedorova T.D., Schaldemose J.L., Knudsen K., Ismail R., Seger A.D. (2021). Regional locus coeruleus degeneration is uncoupled from noradrenergic terminal loss in Parkinson’s disease. Brain.

[70] Persons A.L., Desai Bradaric B., Kelly L.P., Kousik S.M., Graves S.M., Yamamoto B.K., Napier T.C. (2021). Gut and brain profiles that resemble pre-motor and early-stage Parkinson’s disease in methamphetamine self-administering rats. Drug Alcohol. Depend..

[71] Fornai F., Lenzi P., Ferrucci M., Lazzeri G., di Poggio A.B., Natale G., Busceti C.L., Biagioni F., Giusiani M., Ruggieri S. (2005). Occurrence of neuronal inclusions combined with increased nigral expression of alpha-synuclein within dopaminergic neurons following treatment with amphetamine derivatives in mice. Brain Res. Bull..

[72] Butler B., Gamble-George J., Prins P., North A., Clarke J.T., Khoshbouei H. (2014). Chronic Methamphetamine Increases Alpha-Synuclein Protein Levels in the Striatum and Hippocampus but not in the Cortex of Juvenile Mice. J. Addict. Prev..

[73] Klein C., König I.R. (2021). Exploring Uncharted Territory: Genetically Determined Sex Differences in Parkinson’s Disease. Ann. Neurol..

[74] Callaghan R.C., Cunningham J.K., Sajeev G., Kish S.J. (2010). Incidence of Parkinson’s disease among hospital patients with methamphetamine-use disorders. Mov. Disord..

[75] Callaghan R.C., Cunningham J.K., Sykes J., Kish S.J. (2012). Increased risk of Parkinson’s disease in individuals hospitalized with conditions related to the use of methamphetamine or other amphetamine-type drugs. Drug Alcohol. Depend..

[76] Granado N., Ares-Santos S., Moratalla R. (2013). Methamphetamine and Parkinson’s disease. Park. Dis..

[77] Moratalla R., Khairnar A., Simola N., Granado N., García-Montes J.R., Porceddu P.F., Tizabi Y., Costa G., Morelli M. (2017). Amphetamine-related drugs neurotoxicity in humans and in experimental animals: Main mechanisms. Prog. Neurobiol..

[78] Lappin J.M., Darke S. (2021). Methamphetamine and heightened risk for early-onset stroke and Parkinson’s disease: A review. Exp. Neurol..

[79] Curtin K., Fleckenstein A.E., Robison R.J., Crookston M.J., Smith K.R., Hanson G.R. (2015). Methamphetamine/amphetamine abuse and risk of Parkinson’s disease in Utah: A population-based assessment. Drug Alcohol. Depend..

[80] Moszczynska A., Fitzmaurice P., Ang L., Kalasinsky K.S., Schmunk G.A., Peretti F.J., Aiken S.S., Wickham D.J., Kish S.J. (2004). Why is parkinsonism not a feature of human methamphetamine users?. Brain.

[81] Kish S.J., Boileau I., Callaghan R.C., Tong J. (2017). Brain dopamine neurone ‘damage’: Methamphetamine users vs. Parkinson’s disease—A critical assessment of the evidence. Eur. J. Neurosci..

[82] Prince M., Albanese E., Guerchet M., Prina M. (2014). World Alzheimer Report 2014. Dementia and Risk Reduction: An Analysis of Protective and Modifiable Risk Factors.

[83] Lane C.A., Hardy J., Schott J.M. (2018). Alzheimer’s disease. Eur. J. Neurol..

[84] Kelly S.C., He B., Perez S.E., Ginsberg S.D., Mufson E.J., Counts S.E. (2017). Locus coeruleus cellular and molecular pathology during the progression of Alzheimer’s disease. Acta Neuropathol. Commun..

[85] Iversen L.L., Rossor M.N., Reynolds G.P., Hills R., Roth M., Mountjoy C.Q., Foote S.L., Morrison J.H., Bloom F.E. (1983). Loss of pigmented dopamine-beta-hydroxylase positive cells from locus coeruleus in senile dementia of Alzheimer’s type. Neurosci. Lett..

[86] German D.C., Manaye K.F., White C.L., Woodward D.J., McIntire D.D., Smith W.K., Kalaria R.N., Mann D.M.A. (1992). Disease-specific patterns of locus coeruleus cell loss. Ann. Neurol..

[87] Dutt S., Li Y., Mather M., Nation D.A. (2020). Brainstem Volumetric Integrity in Preclinical and Prodromal Alzheimer’s Disease. J. Alzheimer’s Dis..

[88] Hou R., Beardmore R., Holmes C., Osmond C., Darekar A. (2021). A case-control study of the locus coeruleus degeneration in Alzheimer’s disease. Eur. Neuropsychopharmacol..

[89] Betts M.J., Cardenas-Blanco A., Kanowski M., Spottke A., Teipel S.J., Kilimann I., Jessen F., Düzel E. (2019). Locus coeruleus MRI contrast is reduced in Alzheimer’s disease dementia and correlates with CSF Aβ levels. Alzheimer’s Dement.

[90] Liu L., Luo S., Zeng L., Wang W., Yuan L., Jian X. (2013). Degenerative alterations in noradrenergic neurons of the locus coeruleus in Alzheimer’s disease. Neural Regen. Res..

[91] O’Neil J.N., Mouton P.R., Tizabi Y., Ottinger M.A., Lei D.L., Ingram D.K., Manaye K.F. (2007). Catecholaminergic neuronal loss in locus coeruleus of aged female dtg APP/PS1 mice. J. Chem. Neuroanat..

[92] Jardanhazi-Kurutz D., Kummer M.P., Terwel D., Vogel K., Dyrks T., Thiele A., Heneka M.T. (2010). Induced LC degeneration in APP/PS1 transgenic mice accelerates early cerebral amyloidosis and cognitive deficits. Neurochem. Int..

[93] Liu Y., Yoo M.J., Savonenko A., Stirling W., Price D.L., Borchelt D.R., Mamounas L., Lyons W.E., Blue M.E., Lee M.K. (2008). Amyloid pathology is associated with progressive monoaminergic neurodegeneration in a transgenic mouse model of Alzheimer’s disease. J. Neurosci..

[94] Kang S.S., Liu X., Ahn E.H., Xiang J., Manfredsson F.P., Yang X., Luo H.R., Liles L.C., Weinshenker D., Ye K. (2020). Norepinephrine metabolite DOPEGAL activates AEP and pathological Tau aggregation in locus coeruleus. J. Clin. Investig..

[95] Ehrenberg A.J., Nguy A.K., Theofilas P., Dunlop S., Suemoto C.K., Di Lorenzo Alho A.T., Leite R.P., Diehl Rodriguez R., Mejia M.B., Rüb U. (2017). Quantifying the accretion of hyperphosphorylated tau in the locus coeruleus and dorsal raphe nucleus: The pathological building blocks of early Alzheimer’s disease. Neuropathol. Appl. Neurobiol..

[96] Braun D.J., Van Eldik L.J. (2018). In vivo Brainstem Imaging in Alzheimer’s Disease: Potential for Biomarker Development. Front. Aging Neurosci..

[97] Chalermpalanupap T., Schroeder J.P., Rorabaugh J.M., Liles L.C., Lah J.J., Levey A.I., Weinshenker D. (2018). Locus Coeruleus Ablation Exacerbates Cognitive Deficits, Neuropathology, and Lethality in P301S Tau Transgenic Mice. J. Neurosci..

[98] Heneka M.T., Ramanathan M., Jacobs A.H., Dumitrescu-Ozimek L., Bilkei-Gorzo A., Debeir T., Sastre M., Galldiks N., Zimmer A., Hoehn M. (2006). Locus ceruleus degeneration promotes Alzheimer pathogenesis in amyloid precursor protein 23 transgenic mice. J. Neurosci..

[99] Kalinin S., Gavrilyuk V., Polak P.E., Vasser R., Zhao J., Heneka M.T., Feinstein D.L. (2007). Noradrenaline deficiency in brain increases beta-amyloid plaque burden in an animal model of Alzheimer’s disease. Neurobiol. Aging.

[100] Kelly S.C., McKay E.C., Beck J.S., Collier T.J., Dorrance A.M., Counts S.E. (2019). Locus Coeruleus Degeneration Induces Forebrain Vascular Pathology in a Transgenic Rat Model of Alzheimer’s Disease. J. Alzheimer’s Dis..

[101] Heneka M.T., Galea E., Gavriluyk V., Dumitrescu-Ozimek L., Daeschner J., O’Banion M.K., Weinberg G., Klockgether T., Feinstein D.L. (2002). Noradrenergic depletion potentiates beta -amyloid-induced cortical inflammation: Implications for Alzheimer’s disease. J. Neurosci..

[102] Gao Z.-x., Zhang C., Lu J.-c., Zhao X., Qiu H., Wang H.-j. (2021). Pathological methamphetamine exposure triggers the accumulation of neuropathic protein amyloid-β by inhibiting UCHL1. NeuroToxicology.

[103] Zhu Y., Wang X., Hu M., Yang T., Xu H., Kang X., Chen X., Jiang L., Gao R., Wang J. (2022). Targeting Aβ and p-Tau Clearance in Methamphetamine-Induced Alzheimer’s Disease-Like Pathology: Roles of Syntaxin 17 in Autophagic Degradation in Primary Hippocampal Neurons. Oxid. Med. Cell Longev..

[104] Nopparat C., Boontor A., Panmanee J., Govitrapong P. (2022). Melatonin Attenuates Methamphetamine-Induced Alteration of Amyloid β Precursor Protein Cleaving Enzyme Expressions via Melatonin Receptor in Human Neuroblastoma Cells. Neurotox. Res..

[105] Mercan D., Heneka M.T. (2022). The Contribution of the Locus Coeruleus-Noradrenaline System Degeneration during the Progression of Alzheimer’s Disease. Biology.

[106] Kleinridders A., Cai W., Cappellucci L., Ghazarian A., Collins W.R., Vienberg S.G., Pothos E.N., Kahn C.R. (2015). Insulin resistance in brain alters dopamine turnover and causes behavioral disorders. Proc. Natl. Acad. Sci. USA.

[107] West M.J. (2018). Space Balls Revisited: Stereological Estimates of Length With Virtual Isotropic Surface Probes. Front. Neuroanat..

[108] Deniz Ö.G., Altun G., Kaplan A.A., Yurt K.K., von Bartheld C.S., Kaplan S. (2018). A concise review of optical, physical and isotropic fractionator techniques in neuroscience studies, including recent developments. J. Neurosci. Methods.

